# Diseases of the Eye

**Published:** 1899-06

**Authors:** 


DISEASES OF THE EYE. A Handbook of Ophthalmic Practice, for Students and Practitioners. By G. E. DE SCHWEINITZ, A. M., M. D., Professor of Ophthalmology in the Jefferson Medical College; Professor of Diseases of the Eye in the Philadelphia Polyclinic, etc. Illustrated. Third edition. Philadelphia: W. B. Saunders. 1899.

The fact that this handbook of de Schweinitz has had such professional approval as to require three editions within six years, is a more emphatic commendation than it is possible for us to give. The present edition has been fully revised and several new subjects have been introduced. In its improved form, it will continue to hold its place in the esteem of physicians and students, and its continued popularity is assured. A. A. H.

